# Dendrochronology and Isotope Chronology of *Juglans neotropica* and Its Response to El Niño-Related Rainfall Events in Tropical Highlands of Piura, Northern Peru

**DOI:** 10.3390/plants14111704

**Published:** 2025-06-03

**Authors:** Tone Marie Ektvedt, Michael N. Evans, Donald A. Falk, Paul R. Sheppard

**Affiliations:** 1 Department of Geography, University of Bergen, Fosswinchelsgate 6, 5020 Bergen, Norway; 2 Laboratory of Tree-Ring Research, University of Arizona, Bryant Bannister Tree-Ring Building, 1215 E. Lowell Street, Tucson, AZ 85721, USA; mnevans@umd.edu (M.N.E.); prs@arizona.edu (P.R.S.); 3 Department of Geology & Earth System Science Interdisciplinary Center, University of Maryland, College Park, MD 20742, USA; 4 Department of Geosciences, University of Arizona, Gould-Simpson Building 77, 1040 E. 4th Street, Tucson, AZ 85721, USA; 5 School of Natural Resources and the Environment, University of Arizona, Environment and Natural Resources Building 2, Tucson, AZ 85721, USA

**Keywords:** tropical forests, oxygen 18, isotopic analysis, radiocarbon dating, wood anatomy, climate variability, El Niño Southern Oscillation (ENSO)

## Abstract

Tropical trees represent an important potential archive of climate and ecological information, but their dendrochronology based on conventional techniques has been challenging. We conducted a pilot study of the wood anatomy and dendroclimatological potential of *Juglans neotropica* Diels (Juglandaceae), an IUCN Red List species, using 225 radii sampled from 57 trees in Piura (4°55′ S, 79° 56′ W), northern Peru. A total of 112 radii from 40 trees passed quality control and are included in the tree-ring width chronology for this species. *J. neotropica* has demonstrably annual rings, and results are consistent with reports that the species has a dormant period during the dry season, which locally is approximately June–November. Local precipitation is correlated (*p* = 0.10, 1-tailed test) with tree-ring growth, lagged by one year, consistent with other studies of tropical tree species. The age distribution of the sample collection of *J. neotropica* is young and invariant, probably because of selective cutting by local villagers. To supplement ring-width analysis, we conducted the first oxygen isotopic (δ^18^O) and radiocarbon (∆^14^C) analysis for this species on radii from two individuals; results are preliminary given sample size limitations, but consistent with dendrochronological dating, within uncertainties, in all three chronometric analyses. A two-sample composite annually-averaged δ^18^O anomaly data series is correlated significantly with gridded regional growing season (December–May) precipitation (1973/74–2005/06). Qualitatively consistent with simulation of ring width and δ18O, responses to El Niño events are manifested in positive ring-growth anomalies and negative isotopic anomalies following known event years. The combination of tree-ring, radiocarbon, stable isotopic analyses, and the application of sensor and chronological modeling provides a degree of confidence in the results that would not have been possible by relying on any single approach and indicates the potential for further investigation of this and other tropical tree species with uncertain ring boundaries.

## 1. Introduction

Tropical forests remain substantially understudied dendrochronologically relative to their distribution and ecological importance [1,2,3]. Investigations of growth periodicity in tropical tree species have been conducted over the last century [4,5,6,7,8,9], but annual tree-ring chronologies remain rare in the neotropics compared to other biomes [3,10,11,12]. Tropical trees may be problematic for dendrochronology for several reasons: (1) although tropical climates are often hydrologically and phenologically seasonal [13,14], they may lack distinct seasonal variation in temperature, so trees do not always produce visible and reliably annual rings associated with seasonal thermal dormancy [15]; (2) perhaps because of hydrologically mediated seasonal dormancy ([16] and references therein), tropical trees may form false rings and individuals may present missing rings, making the establishment of proxy records with absolute chronologies more difficult [17,18,19]; (3) species that produce long time series can be difficult to locate and sample; and (4) trees may be disturbed by human activities, making the climate-growth signal difficult to interpret [20,21,22,23,24]. Identifying species useful for tropical dendrochronology thus remains a challenge in this field [23,25], although progress has been made for high elevation sites and species in Peru [19,26,27,28], southern Ecuador, northern Argentina and Chile, and elsewhere [1,29,30,31,32,33,34], as well as the Amazon Nordeste region [14].

Seasonal and interannual variation in climate regulates tree growth and demography in tropical regions [27,35,36]. El Niño/Southern Oscillation (ENSO) events occur irregularly approximately every two to seven years [37], and their effects on tropical regional climate are pronounced [38]. Northern coastal Peru is often a focal point for initiation of large storm events and high precipitation periods during El Niño events, which can cause precipitation anomalies of up to 60 times the climatological mean [39,40,41,42,43]. This makes northern Peru especially relevant for studies of ENSO effects on terrestrial climate ([27] and references therein).

Tree-ring studies on *Prosopis* spp. [42,44,45,46] and *Bursera graveolens* [42] in northern Peru and Chile indicated an El Niño growth increase in lowland areas. Investigations of *Prosopis* spp. in northern Peru concluded that lowland tree species respond strongly to precipitation variation [44]. Expansion of dendrochronological work into regional highland tree species, including the establishment of multi-species networks, might complement these studies by improving cross-site replication and dating precision, producing generalized environmental growth response functions for the Peruvian dry forest, and strengthening spatial and temporal climate reconstruction efforts and hydroclimate response patterns across species [27,47,48,49,50].

Radiocarbon dating can be used as a supplemental method in tropical dendrochronology to determine tree ages and/or support the determination of annual rings [17,19,51,52,53,54,55,56,57], or to construct isotope chronologies for tree species without visible annual rings [54,58]. During the late 1950s and early 1960s the concentration of radiocarbon (^14^C) doubled in the Earth’s atmosphere due to atmospheric nuclear weapons testing [51,59,60]. Tropospheric ^14^C subsequently decreased with the flux of carbon into biological, land surface and ocean reservoirs [61]. Since living plants assimilate the concurrent atmospheric radiocarbon concentration, so-called ‘bomb radiocarbon’ can be used to date recent organic material, such as wood from living or recent trees [19,51]. Radiocarbon dating has been used in tropical trees to determine growth rates [62] and to investigate whether visible wood anatomical features are annual growth rings [17,19,52,53,55,56] or not [54,57,58,63]. Radiocarbon analysis has also been used to validate analyses of tree-ring growth response to climatic events, including flooding [52], rainfall events [17,64], or ENSO events [55,65], and to develop isotope age models [58,64,65].

Stable isotope techniques permit advances in tropical dendrochronology beyond those possible from visual tree-ring width analysis alone (e.g., [15,25,65,66,67,68,69,70,71,72]). Because wood cellulose tends to be stable at surface conditions (e.g., [73]), and because the environmental controls on its stable isotope composition are relatively well understood (e.g., [74,75,76]), carbon, oxygen and hydrogen isotope records from cellulose can be developed [77]. The oxygen isotopic composition of tree cellulose reflects air temperature, relative humidity, and the isotopic composition of source water [58,78,79], the latter determined by temperature path history of atmospheric moisture [80,81], as well as leaf-level evaporative effects and tree physiological processes [76,82,83]. In our study area (montane western South America), temperature is relatively constant through the year; thus, it has been proposed that variations in the oxygen isotopic composition of wood cellulose primarily reflect variations in the amount of precipitation [15,65,66,67] and/or the temperature path history of the precipitating moisture [71,84]. Based on these empirical studies, as well as forward modeling ([76,85] and references therein), we expect that, for tropical locations with high and constant temperature, high humidity, and pronounced seasonal and/or interannual variations, higher precipitation amounts will be expressed as lower cellulose δ^18^O values, and lower precipitation amounts will be expressed as higher cellulose δ^18^O values. For sub-annually resolved sampling, we can use the hypothesized annual cycle in cellulose δ^18^O to develop a relative age model by assigning periodic isotopic maxima to the beginning and end of the climatological dry season (e.g., [65]).

*Juglans neotropica* Diels [86] (Juglandaceae: walnut family), common name: ‘nogal’ is a highland species first described by Diels [87]. It is found primarily in dry to humid lower montane forests between 1000 m and 2800 m elevation, and ranges from northwestern Venezuela and northern Colombia through Ecuador and Peru [88,89,90]; it is also used for forestry in New Zealand [91]. *J. neotropica* is valued as a timber, medicinal, and nutritional resource; a bark extract is used to dye wool (personal communication; Luis Gongolo Córdova; carpenter and owner of *J. neotropica* trees in Frias; [90,92,93]). *J. neotropica* is a conservation priority because of its extensive use and documented decline [93,94,95,96] and is on the Red List of endangered species [97], due in part to over-exploitation for timber [90,98]. Because of its wide range over a region affected directly by ENSO in the eastern Pacific Ocean, and its socioeconomic importance, *J. neotropica* is a prime candidate for dendrochronological and dendroclimatological analysis [19,27].

Relatively few dendrochronological studies have been published for the genus *Juglans*, but its dendrochronological importance and potential have been recognized [3,19,27]. *Juglans* are generally diffuse-porous [99,100], meaning that the vessels are of approximately equal diameter throughout the ring [101]. *J. australis* cross-dates both within and among trees [11,29,102,103,104]. Wood growth in tropical areas generally starts soon after the beginning of the rainy season, and in deciduous species growth stops at the end of the rainy season [105]. *J. neotropica* is reported to be dormant during the winter months [91], which normally extend from June to August. Villalba et al. [29] indicate that the growth period of *J. australis* lasts from September to April/May in Argentina and western Bolivia. However, there is disagreement as to whether *J. neotropica* is evergreen [95,96,106] or deciduous [89,91,99].

We present dendrochronological, oxygen isotopic and radiocarbon analyses in this pilot study to establish a preliminary regional growth chronology for *J. neotropica* from the Pacific slopes of the Andean foothills of northwestern Peru, with reference to effects of El Niño on growth patterns. We couple ring studies with isotopic analysis and radiocarbon dating to strengthen inferences about the timing of tree growth in tropical locations, such as our northern Peru study area. In addition, we calibrate the isotopic signature of climatic variations associated with the regional expression of El Niño activity, the first step toward using the species as an archive of paleoclimatic indicators in this key geographic area. In northwest coastal tropical Peru, we expect an annual precipitation cycle to produce anatomically distinct annual ring structures associated primarily with moisture availability [15,42], for which cross-dated chronologies should be in agreement with radiocarbon dates from hypothesized 1970s–1980s wood. Although seasonal dormancy has not been observed directly, we postulate that growth occurs in *J. neotropica* within the rainy season, based on studies in closely related taxa (e.g., [29]). On interannual timescales, we expect growth of *J. neotropica* to be controlled by variation in precipitation amount, as found for *Juglans australis* [103] and other tropical tree species [31,44,103,107,108]. Specifically, during ENSO warm phase conditions, when precipitation is unusually high, we expect increased ring widths (e.g., [16,42]) and lower oxygen isotopic composition [15,85] during or shortly after such events. Our specific research questions are as follows:

1. Can *J. neotropica* be cross-dated [109] accurately using visible wood anatomical features?
2. Are ring-based age estimates consistent with radiocarbon and stable isotopic estimates?
3. Are variations in growth rates and oxygen isotopic composition of *J. neotropica,* and their relationship to regional precipitation variations and ENSO, consistent with that demonstrated for other species?

## 2. Data and Methods

### 2.1. Study Site

**Figure 1:**
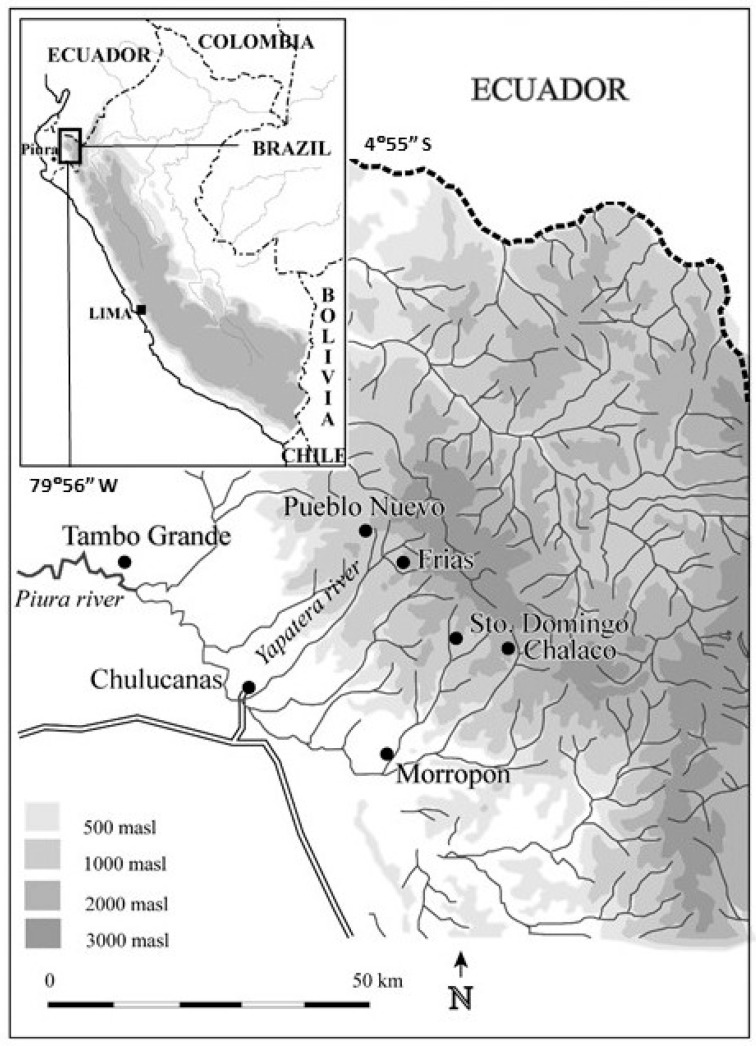
Study area and locations of sampling and climate stations.

Field work was conducted in 2005 and 2007 in the north Peruvian villages of Frias and Pueblo Nuevo (04°55′ S, 79°56′ W, elevation 1765–1870 m a.s.l.), Piura Province, Peru (Figure 1). The study sites are classified as ‘premontane dry forest’ (‘bosque seco premontaño’ [110]. Premontane dry forest occurs in tropical areas along the lower slopes of mountains where there is a long dry season, alternating with a 5–6 month precipitation period. The study area consists of variably dense forest, with tree canopy coverage of 40–70% on soils of mostly sand, silt and clay overlying Cretaceous intrusive units (diorites, granodiorites, tonalites, granites) and volcanic units; landslides and seepage erosion are frequent following periods of higher precipitation [111,112]. The combination of elevation and latitude produces relatively warm days and cool nights (see below; [112]). The study area supports rich species diversity; typical tree and bush species include *Juglans neotropica* (nogal), *Persea americana* (palto) and other *P.* spp., *Erythrina edulis* (pajul), *Baccharis latifolia* (chilca), *Nectandra reticulata* (paltaguiro), *Rubus* spp. (blackberry), *Croton* spp., *Tibouchina* spp. and *Heliotropicum* spp. [112].

Forest canopy height in these areas is approximately 15–25 m, but trees could probably grow higher in areas without human extraction of forest resources. Toro and Roldán [90] document a tree height of *J. neotropica* in South America of 15–48 m, with deep roots that can reach more than three meters in depth. Small-scale subsistence agriculture, cattle breeding and forestry are the main activities of the inhabitants [112]. Households are clustered, with agricultural areas spread out in the local area. To minimize risk of pests and disease, several species are cultivated together on a small scale, where farmers often change cultivated species from year to year. Areas of natural vegetation are mainly in areas that are difficult to cultivate, relatively far from villages, or as natural boundaries and windbreaks between small-scale agricultural plots.

**Figure 2:**
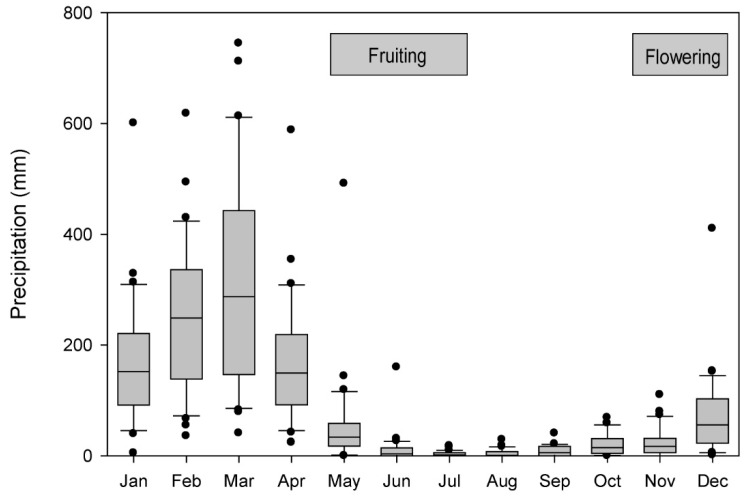
Monthly maximum, mean and minimum precipitation (mm) in Frias, Peru for 1963–2003. (Source: [113]). Standard box plot convention is followed (box encloses 25th–75th percentile with median line; outer bars are 10th and 90th percentiles; outliers are shown as dots). Horizontal bars indicate the mean periods of flowering and fruiting of *J. neotropica* [93].

Precipitation in Frias is generally received from December to May/June (5–6 months) (precipitation data from Frias 1963–1992 and 2002–2003 [113], with a dry season from May/June to November (Figure 2). Because local precipitation data from Frias are missing for the periods January 1993–December 2001 and January 2004–December 2007, we compiled additional precipitation data from Santo Domingo (05°02′ S, 79°52′ W, 1475 m a.s.l.), 14 km away [113] SENAMHI, 2025; Figure 1). We analyzed historic annual precipitation data, designating the water year from September to August. Santo Domingo and Frias precipitation data series are correlated significantly (*r* = 0.82, df = 31, *p* < 0.05) for the period 1963/64–1991/92, 2001/02–2002/03 for which data are available for both stations. During the El Niño event of 1982/83, Frias experienced five times more precipitation (above 3400 mm rain from September 1982 to August 1983) than the mean annual precipitation of 648 mm (data range: 1964–2007, excluding the El Niño events of 1982/83 and 1997/98). During the same El Niño event, Santo Domingo experienced nearly seven times more rain (more than 3100 mm from September 1982 to June 1983) compared to a mean annual precipitation period of 533 mm (data range: (1964–2007, without the El Niño events of 1982/83 and 1997/98).

Temperature data at the immediate study site are unavailable, but Sausal de Curucan, 27 km distant at an elevation of 980 m a.s.l., had a yearly mean temperature of 22.5 °C, and a mean annual range of 17.1–29.3 °C during 1972–1996 [113]. Based on the moist Environmental Lapse Rate (ELR) and the International Standard Atmosphere (ISA; [114]), mean temperature decreases at a moist adiabatic rate of 6.49 °C/1000 m; using this correction, we estimate the yearly mean temperature in Frias to be 16.7–17.4 °C, which is consistent with other estimates of the growing season temperature range for *J. neotropica* of 16–28 °C [115] and 11–19 °C [116]. Mean interannual variation in temperature is about 4 °C, although records for 1972–1996 indicate a temperature range of 14–30 °C [113]. Based on these data, we infer that temperature variations in Frias are not likely to limit growth for *J. neotropica*.

### 2.2. Field Sampling

We sampled 225 radii of *J. neotropica* from 56 trees using Haglöf increment borers of 5.1 mm diameter/600 mm length and 4.3 mm diameter/200 mm length, following standard dendrochronological methods [109]. One additional cross-sectional sample was also recovered from a cambial active stump (adventitious cambial growth) of a previously felled tree (total N = 57). Because sampled *J. neotropica* produced colored fluids when cored, it was difficult to identify the intersection of cores with, or proximity to, the pith in the field. We sampled four radii from each tree, one from each cardinal direction. Cores were stored in paper straws and air dried, then stored in a freezer to prevent disease or pathogen transport. Subsequent sample preparation and analysis were performed at the Laboratory of Tree-Ring Research, University of Arizona, Tucson, AZ, USA.

### 2.3. Wood Anatomy

We prepared microtome slices of two radii from two *J. neotropica* trees to examine wood structure. We first let the wood soak in three parts water to two parts glycerin to soften samples for cutting. We then used a sliding microtome to cut slices as thin as possible without breaks; this procedure yielded slide thicknesses of 50–70 µm. We mounted the sections on glass slides and took images of each slide using a light microscope (Nikon SMZ-U; Nikon, Tokyo, Japan) equipped with a digital camera (Olympus E-330; Olympus, Tokyo, Japan). We used the microtome slides to complement traditional visual investigation of tree samples under a dissecting microscope to investigate the cell structure and wood anatomy of *J. neotropica*.

The determination of annual ring boundaries in diffuse porous species can be based on a variety of characteristics (e.g., [23]). We examined pore size, wood density, presence of parenchyma, and differences in vessel diameter and color from the beginning to the end of the growth band. We classified our observations as indicating growth rings in *J. neotropica* if (a) they were present in all samples, (b) the ring was uninterrupted by vessel cells going through the parenchyma line, and (c) there was a clear discontinuity of late wood cells and early wood cells. We noted differences in ring sequences in radii within and among sampled trees.

### 2.4. Dendrochronological Sample Preparation, Tree-Ring Analysis and Modeling

We sanded samples with progressively finer sandpaper to grit size 1200. We visually cross-dated radii from 57 trees to the innermost ring of the radius. Of these, we excluded 17 trees that were younger than 12 years of age (1995/96) because juveniles can be more prone to competition stress, geomorphic and wind stress that can complicate the climatic signal [101], leaving 112 radii (*n* = 40) for analysis. We measured annual rings to the nearest ± 0.001 mm under a binocular stereoscope with a Velmex measuring machine connected to a computer [117]. We measured rings perpendicularly to a line tangent to the curve of the ring to maintain measurement accuracy despite variations in the coring angle [118].

We cross-dated and checked ring data using the skeleton plot method [119] and used the computer program ARSTAN (v.6; [120]) to detrend each tree series for the period 1963–2006. We detrended tree series with a negative exponential curve or linear regression to minimize non-climatic growth influences, such as competition, tree age and diameter, forest disturbances and defoliation [121]; indices were computed by division. We calculated correlation statistics for the years that were common to the trees used in analysis (1977–2006) (Table 1b).

We used the process-based model VS-Lite [122] to explore the mechanistic relationship of tree growth to climate variables (Supplementary Information). VS-Lite provides a realistic nonlinear multivariate tree growth model based on the principle of limiting factors [123]. We generated cumulative age-growth plots by integrating annual ring widths over time, to examine growth trends and variation among trees with varying years of origin [124,125]. We plotted and compared cumulative actual and normalized diameter growth to both calendar and normalized age for each tree in the analysis set.

### 2.5. Oxygen Isotopic Analysis

#### 2.5.1. Sample Preparation and Data Analysis

We chose one radius from each of two trees (JN37-1W and JN41-2N) from which to analyze oxygen isotopes and develop both an age model and analysis of interannual variations. Trees for this analysis were selected based on their potential for time series length, absence of breaks or twists, and an intersection of the core with the tree pith. One radius (JN41-2N) was sanded with sandpaper to grit size 1200; the other (JN37-1W) was left un-sanded for subsequent radiocarbon analysis to avoid cross-contamination across the steep bomb radiocarbon curve or through intraseasonal oxygen isotopic variations [126].

We used a rotary microtome to sample increment cores perpendicular to the sampled axis of growth, at thicknesses ranging between 0.15 and 0.30 mm per slice, accumulated into sequential independent samples each of about 1 mg wood [15,127]. Alpha-cellulose was extracted using a modified Brendel technique [128]. We wrapped 300–350 μg samples of alpha-cellulose in silver capsules and converted them to carbon monoxide (CO) at ≳1500 °C using a Costech High Temperature Generator (Milan, Italy) [129] and passed the CO to a Thermo Finnigan Delta Plus XP mass spectrometer (Thermo Fisher Scientific, Waltham, MA, USA) via Conflo II open split interface for isotopic analysis. Data were quality controlled for chromatographic evidence of incomplete sample conversion and insufficient sample amplitude (<1V at M44 collector), and trend-corrected using periodic in-run working standard alpha cellulose and monitoring gas measurements. The 1-sigma measurement precision, based on replicate homogeneous working standard alpha cellulose (Sigma Alpha Cellulose (Sigma-Aldrich, St. Louis, MO USA), assigned δ^18^O = 31.0‰, relative to the V-SMOW reference standard), was ±0.3‰. We analyzed a total of 2184 isotopic samples (1315 from radius JN37-1W and 869 from radius JN41-2N).

To age model the δ^18^O data independently from wood anatomical and dendrochronological analyses, we worked backwards from the known collection date in May 2007 to assign periodic isotopic maxima to December and May of prior years, based on precipitation records and following the oxygen isotope chronometric hypothesis (Section 1; see [15,65]). We then linearly interpolated to obtain date estimates for isotopic data within each growing season December to May, and assumed growth hiatus during the dry season (between May and December). The age-modeled series were then averaged across growing season and core series to form a composite δ^18^O data series at annual resolution.

To model the chronological uncertainty in these data, we applied the banded age model (BAM) of Comboul et al. [130], which assumes a cumulative error of omission or addition of annual growth increments as a function of a specified error rate. Based on the radiocarbon and oxygen isotope based chronometric results and the method of age model development, for which either sampling or date assignments are relative to a known distance from bark or date of collection, we specify a Poisson distribution of errors and an error rate of 10%. We applied the same model to the TRW chronology with an error rate of 5%.

#### 2.5.2. Isotopic Modeling

Interpretation of the annually resolved composite δ^18^O was facilitated by comparison with simulated δ^18^O, in which the simulated values were those predicted using the model of Evans [85], updated to sample forcing uncertainty [131]. In contrast to linear or multivariate linear modeling, the isotopic model reflects the combined influences of temperature (T), precipitation (P) and humidity (q) variations at monthly resolution, based on eco-physiological and stable isotope modeling [75,76,85]. It thus provides a basis for comparing observed δ^18^O to local climate variations. We used the CRU TS4.08 [132] gridded monthly Dec-May precipitation amount, temperature and specific humidity as modeling inputs. We averaged the simulated δ^18^O to December–May values for comparison with the observed δ^18^O composite. Parameters used in the simulation exercise were as in Table 1 and Table 2 of [85]), with a prescribed uncertainty of 20% for T, P and q inputs.

### 2.6. Correlation Analysis

We examined the relationships between the tree-ring width of *J. neotropica* and precipitation, and between isotope composition and precipitation, using correlation functions. We tested correlations between standard tree-ring width chronology and mean isotope values per growing season with local growing season precipitation records from Frias and Santo Domingo. Because we found that the tree-ring widths (TRWs) were serially autocorrelated (Table 1), we used the effective degrees of freedom (edf) as $\left(n\right)\left(\frac{1-AR1}{1+AR1}\right),\;$where *n* = number of observations and *AR1* = first order autocorrelation coefficient ([133] and references therein). We used a one-tailed *t*-test because we did not hypothesize a negative slope for the regression of TRW on precipitation, a positive slope for the regression of cellulose δ^18^O on precipitation, or a negative slope for the regression of simulated on observed cellulose δ^18^O. Because isotopic composition of precipitation in equatorial regions is driven by hydrological processes at regional scales [133,134], we also used gridded precipitation data (CRU T4.08; resolution 0.5° × 0.5°), based on regional interpolation and quality control, from 82.25–77.75° W to 7.25–2.75° S, using mean December–May precipitation in the correlation analysis in addition to station data [135]. The time series used for the gridded precipitation data were 1959/60 to 2005/06.

### 2.7. Radiocarbon Analysis

To validate the isotopic and dendrochronological age models of *J. neotropica,* we selected five wood samples from JN37-1W for radiocarbon analysis. Based on the oxygen isotopic age model, radiocarbon samples were selected from core intervals corresponding to 1–2 years each and spanning the full estimated age of the cored tree. Samples containing 4–5 mg of wood were treated following the procedure described in [136] to extract cellulose [128]. Treated samples were submitted to the NSF-Arizona Accelerator Mass Spectrometry (AMS) Laboratory, University of Arizona, for combustion to CO_2_, purification, and high-precision ^14^C analysis; calibrated ages were developed using OxCal version 4.1 [137,138]. Results from radiocarbon analyses, including measurement uncertainty (two year sampling age resolution) were fitted to the observed Southern Hemisphere bomb radiocarbon dataset [61], with the constraint that samples must be ordered in age as they are ordered in depth toward the tree core pith [137]. Age ranges reported are for the two sigma ranges estimated by OxCal 4.1 [138], which indicates a 95.4% probability that the correct calendar date falls within this range [139].

## 3. Results

### 3.1. Wood Anatomy of J. neotropica

**Figure 3:**
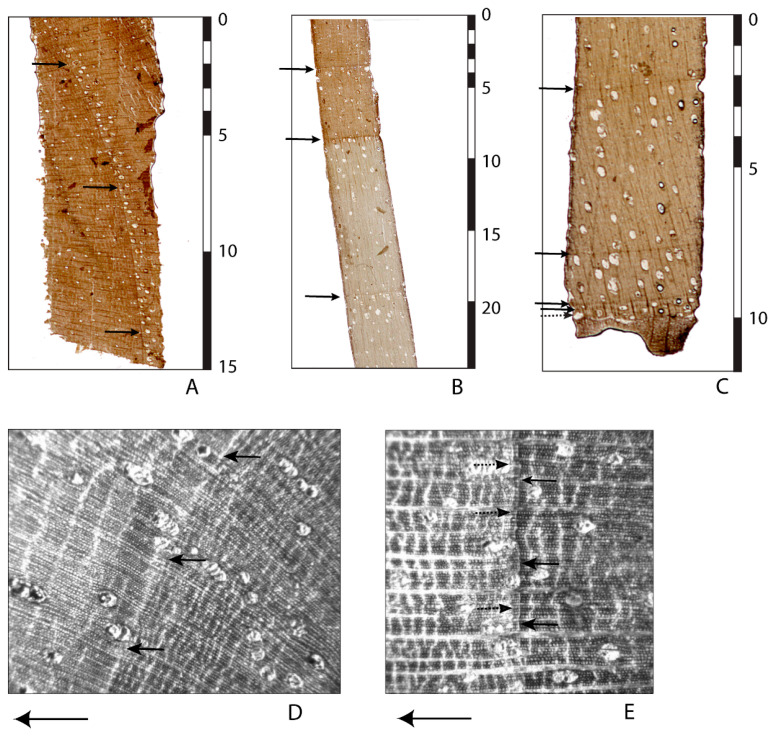
Microscope images of *J. neotropica* microtone slices showing ring boundaries (arrows) near the core (**A**), in the transition zone from heartwood to sapwood (**B**) and near the bark (**C**). A piece of the bark is seen in the lowermost part of (**C**), indicating the direction of growth from top to bottom. Scale bars in mm. (**D**,**E**) Wood structure images. All photos by T.M. Ektvedt.

Once samples are prepared in the laboratory, heartwood and sapwood in *J. neotropica* can be differentiated by coloration. Heartwood tends to be dark grey–brown, with some lightening towards the end of the growing season, whereas sapwood tends to be a more uniform creamy yellow (Figure 3B). We defined ring termination by a rapid narrowing of the parenchyma cells to produce a thin dark line, usually about two to four cells wide, tangential to the direction of growth (Figure 3A–C). Parenchyma cells forming the sharp boundary in heartwood can be difficult to discern without extensive preparation of the core surface (Figure 3D). However, an increased number of vessels in the earlywood, often in clusters, and an increased number of wood rays tangential to the direction on latewood growth can also be used to identify a growth boundary (Figure 3A,D). Ring boundaries within the sapwood are visible as darker bands than those that typically occur in the heartwood.

### 3.2. Tree-Ring Chronology of J. neotropica

Our samples showed large variability in ring width among trees. Within a single radius, the mean range factor between smallest and largest ring is 26, but the difference could be as extreme as 1:233 (0.074 mm: 17.274 mm). Mean minimum ring value among samples is 0.84 mm, while mean maximum value is 10.41 mm. Among trees, mean ring width and standard deviation is 4.87 ± 1.49 mm (min among all trees 1.67 mm/yr, max 8.02 mm/yr). Although we cannot eliminate the possibility of missing rings, cross-dating analyses did not indicate dating errors in the ring width chronology.

For the common interval time span (1977–2006), mean correlation across 28 radii from 17 trees of *J. neotropica* is high (0.76), indicating relatively strong within-tree consistency in growth variations around the tree circumference (Table 1b). However, the correlation of all radii compared to the sample mean is relatively low (0.18), and mean inter-tree correlation is near zero, indicating a weak inter-tree correlation (*r*-bar = 0.176).

**Figure 4:**
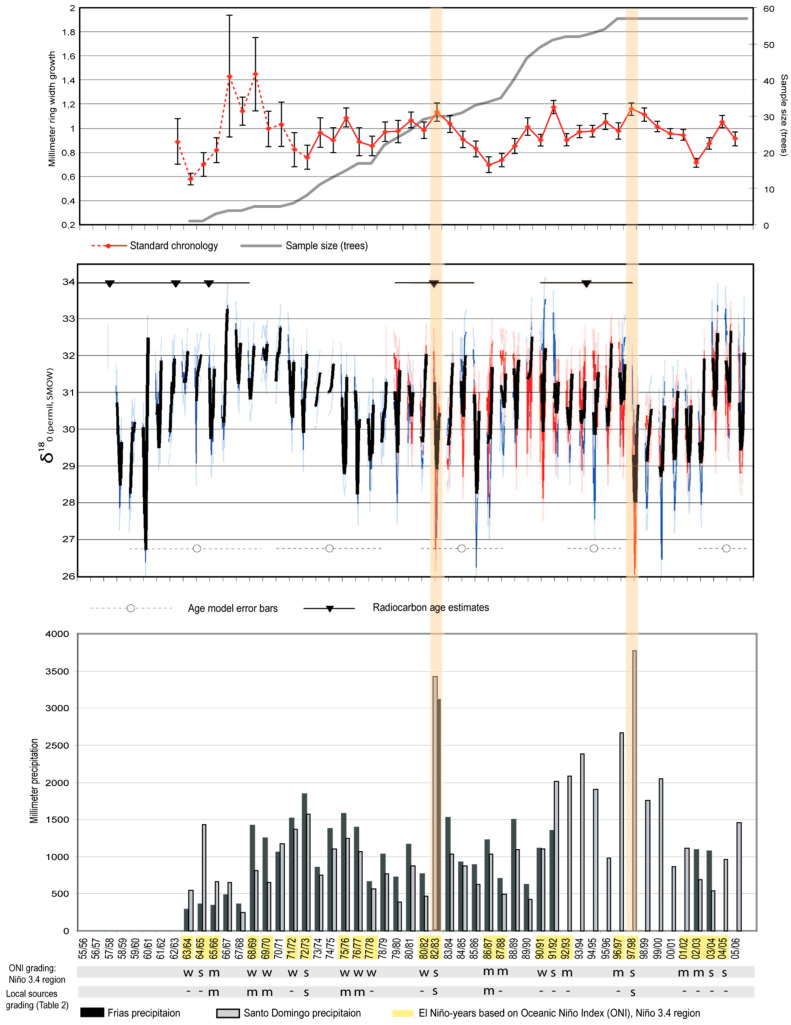
(**Top**) *J. neotropica* standard chronology from 1962/63–2005/06 compared to sample size (number of trees, grey line). The bars on the standard chronology are ±1 standard error of the standard chronology among the samples. Chronology values from 1962–1971, which may not be reliable due to small sample size, are indicated as a dashed line. (**Middle**) Age-modeled oxygen isotope records of JN37-1W (blue) and JN41-2N (red), with monthly-interpolated mean values in black, and radiocarbon age estimates (see Table 2) plotted as nablas with 2s error bars (top). Bottom lines with circles are error bars by decade centered on the decade midpoint: 2000s ± 2 years, 1990s ± 2 years, 1980s ± 3 years, 1970s ± 4 years, 1960s ± 5 years. (**Bottom**) Precipitation at Frias (black bars) and Santo Domingo (grey bars). El Niño years (yellow shading on years) are based on the Oceanic Niño Index [142], with event intensity grading (weak (w), moderate (m), strong (s) and no info (-) (Table 3). The two strong El Niño-events that resulted in precipitation of more than 3000 mm are shaded for comparison across panels.

The total set of measured tree samples from 1963/64–2005/06 covered few trees in the early period (especially 1963/64–1973/74) (Figure 4, top), resulting in relatively few reliable ring-width values during this early period. The earliest rings show a period of larger radial growth compared with rings in the later stage of the chronology, probably reflecting inherent variability due to small sample size during this period, but possibly also reflecting more vigorous growth rates in younger trees [101,140]. Sample size by year (Figure 4, top) reflects only the innermost measured rings and not actual tree ages, since some cores were an unknown number of years from pith. Using pith estimators [141], we estimated that the mean years to pith was 2.1 years (maximum 5 yr). Some chronology values from 1962 to 1971 may not be reliable due to low sample size (<10 samples). The resulting *J. neotropica* standard chronology provides a record of tree ring growth of more than 30 years with sufficient sample size (Figure 4, top).

### 3.3. Stable Isotopic and Radiocarbon

Oxygen isotopic analyses (Figure 4, middle) and age modeling revealed a quasi-cyclical climatic signal associated with the stable isotopic age model (§2.5), with interannual variation. The oxygen isotopic age model indicated that JN37-1W dates to 1958 ± 5 years, with uncertainty based on differences between plausible age models (Figure S1); this is consistent, within error, with both ring width and radiocarbon age estimation (Figure 4: middle section: blue curve). The isotopic interpretation of JN41-2N (Figure 4: middle section: red curve) was dated to 1978 ± 4 years, consistent within age model error with the ring width measurement of the same sample. Radiocarbon results (^14^C) of the five samples taken from the *J. neotropica* radius JN37-1W (Table 2) bracket the ages estimated by dendrochronological and isotopic analysis, given uncertainties in the age model estimates (Figure 4, middle). Our fraction of modern radiocarbon (F^14^C) values revealed an age range from zero (expected age) to eleven years compared to our expected age model. Our youngest radiocarbon sample (sample 5) had a younger calibrated age than expected, while our oldest sample (sample 1) was older than expected.

### 3.4. Correlation of Tree Growth with Local and Regional Climate

Growth modeling with VS-Lite confirmed that ring growth is regulated primarily by soil moisture (*r* = 0.31, edf = 8, *p* = 0.07, one-tailed test; Table 4; Figure S3, Supplementary Information). Two particularly strong El Niño events occurred during the period of analysis, in 1982/83 and 1997/98. Other strong El Niño events occurred in 1991/92 and 2003-05 [142] (Table 3; Figure 4). During the period with more than ten sampled trees (1973/74–2005/06), we found a generally positive (but non-significant) relationship between ring width and gridded precipitation (*r* = 0.25, *p* = 0.10, edf = 14, one-tailed test); Table 4). For the same period, correlation between the 2-sample composite δ^18^O and gridded precipitation is slightly less likely to arise by chance (*r* = −0.31, *p* = 0.06, edf = 12, one tailed test). Correlation between composite δ^18^O and median simulated δ^18^O [85] is significant (*r* = 0.32, *p* = 0.05, edf = 12, one-tailed test) and not sensitive to forcing uncertainty. Correlation between composite δ^18^O and ring width is nonsignificant (*r* = −0.21, *p* = 0.15, edf = 12, one tailed test). Correlations (Table 4) of observed TRW and δ^18^O with the NINO12 SST anomaly index (0–10° S, 90–80° W) are 0.34 and –0.35, respectively, significant at *p* = 0.04; however, when age model uncertainty is sampled, significance is preserved for TRW (95th percentile confidence interval > 0), but not for δ^18^O (Figures S2 and S3).

**Table 3:** El Niño events affecting climate in Peru from 1950 to 1998 classified by [143] as moderate (M) and strong (S).

Period	Strength	Source
1951	M	[41,144]
1953	M+	[41,144]
1957/58	S	[41,144]
1965	M+	[41,144]
1968/69	M−	[41]
1972/73	S	[41,144]
1976	M	[41,144]
1982/83	S+	[41,113,144]
1987	M	[41,143]
1997/98	S+	[113,144,145]

**Table 4:** Correlation of tree ring width (TRW), and oxygen isotopic composition (δ^18^O) and simulated δ^18^O [85] with nearest grid point precipitation (P) from CRU TS4.08 [134] and NINO12 sea surface temperature (SST) index [142].

Correlated Variables	*r*	Student’s *t*	EDF	*p*
P, TRW	0.25	1.35	14	0.10
P, observed δ^18^O	−0.31	−1.71	12	0.06
Observed δ^18^O, simulated δ^18^O	0.32	−1.74	12	0.03
Observed δ^18^O, TRW	−0.21	−1.09	12	0.15
Observed TRW, NINO12 SST index	0.34 (0.04, 0.37)	1.85	12	0.04
Observed δ^18^O, NINO12 SST index	−0.35 (−0.39, 0.44)	−1.96	12	0.04

EDF = minimum (across correlated variables), estimated degrees of freedom [(1 − *r*_1_)/(1 + *r*_1_)] × N; *r*_1_ = AR(1) coefficient; *p* = significance (one-tailed test) of the correlation coefficient given EDF. NINO12 SST index = Dec–May mean climatological SST anomaly for the area (0–10° S, 90° W–80° W; CPC 2025 [145]). For the correlations with NINO12, values in parentheses are the 2.5th and 97.5th percentile values considering age uncertainty estimates, following [130], with a Poisson distribution and annual growth error rates of 5% and 10%, respectively.

### 3.5. Cumulative Tree Growth

**Figure 5:**
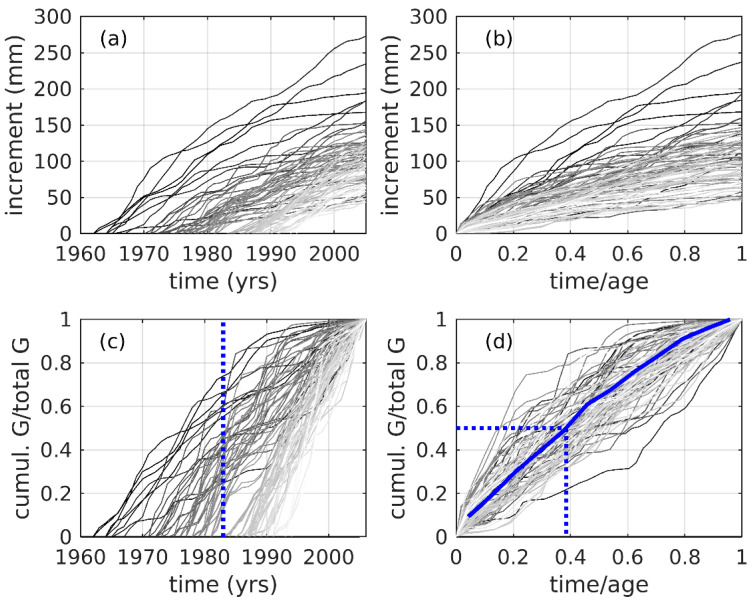
Cumulative growth analyses for study samples of *J. neotropica*. (**a**) Cumulative incremental growth (mm) vs. time for 83 increment series in the *J. neotropica* chronology reported in this study. Darker (lighter) greyscale indicates longer (shorter) time interval spanned per increment core. (**b**) As in (**a**), except for time normalized to the length of each increment series, respectively. (**c**) As in (**a**), except for cumulative growth normalized by total growth of each series, respectively. The vertical dashed line indicates the 1982/83 El Niño event. (**d**) As in (**c**), except for time normalized by length of each increment series, respectively. Blue curve is the median of the median normalized growth-time values, with all normalized cumulative growth series interpolated to a common 12-point linear normalized timescale. Dashed lines trace the values of the median normalized cumulative growth (0.5), reached at 0.39 of the median normalized interpolated age.

Sampled trees were recruited over a 33-year period (1963–1995 inclusive), with the majority recruited in the 1980s and 1990s (Figure 5a, Table 1a). Trees that were recruited during the early portion of the record (1960s–1970s) had higher sustained growth rates than those recruited later (Figure 5b). The 1982/3 EN event is notable as a period of accelerated growth among many of the sampled trees (dashed line in Figure 5c). Plots of normalized growth as a function of normalized time (Figure 5d) indicate that trees achieved their median total growth after only 39% of total time, with the growth curve downwardly inflected, indicating a declining growth rate.

## 4. Discussion

### 4.1. Can J. neotropica Be Crossdated Accurately Using Visible Wood Anatomical Features?

Some authors have described the wood anatomy and the appearance of *J. neotropica* [92,93,94,96,116,146,147,148,149] but could not determine if the observed growth bands were annual or seasonal. Ref. [99] claims that the genus *Juglans* forms annual growth rings, but did not mention *J. neotropica* specifically. The growth rings were seen as a ‘thin layer of flattened fibers and a uniseriate row of parenchyma cells on outer margin’ [99]. Ref. [100] found terminal parenchyma present in *J. neotropica* that they identified as growth rings. However, [150]’s work on wood anatomy of the genus *Juglans* claimed that ‘growth rings are distinct in the temperate species and generally lacking in the tropical species’. Refs. [19,151,152] later concluded that *J. neotropica* had annual rings.

Our results are in accordance with [19,100,151,152], who found annual growth rings. Our study thus contributes to the literature confirming that growth rings can be detected in seasonally dry tropical species. Interannual differences in ring width were observed in the samples of *J. neotropica* (§3.1). To our knowledge, no other studies have reported similar magnitude growth differences in tropical *Juglans* spp.; we cannot evaluate whether such interannual differences are common in the region. Mean ring width of all rings in *J. neotropica* was 4.87 mm, similar to mean ring width of 5.29 mm in *Bursera graveolens* [42] (Rodríguez et al., 2005), which also occurs in the region, but not *Prosopis pallida* [44] (mean ring width of 0.93 ± 0.620 mm). Ref. [30] found that different tropical tree species in the same region can vary from 0.6 mm year^−1^ to 5.7 mm year^−1^. The cumulative radial growth rate was highest during the early years for most trees in our sample (Figure 5d).

The 112 radii from 40 trees of *J. neotropica* in this study suggest dendrochronological potential, beginning with a coherent common signal (*r* = 0.76 for increment cores within the same tree) for this species at this site. However, the low common signal among different trees represents a challenge to dendrochronological reconstruction and merits additional sampling and analysis. Low among-tree correlation may reflect the extensive human modification and use of *J. neotropica* stands; juvenile trees can also be more prone to ecological factors, such as competition, geomorphic events, and wind stress, which can complicate a purely climatic signal [101]. In the effort to obtain a sufficiently large sample size, we sampled both open and closed canopy stands, which may further contribute to variability in the resulting ring width chronology. Refs. [19,27] found high intercorrelation among radii of *J. neotropica* in studies conducted in areas with less human impact, which may indicate that the selection of a study site with less human impact is important. Ref. [90] (and references therein) confirm that *J. neotropica* grows well under conditions of moderate disturbance, such as in agroforestry systems and areas with soils degraded by mining activity. However, if *J. neotropica* could also be sampled in areas with less human impact, longer and more accurate chronologies may be developed.

Accurate precipitation data were scarce at this site, and local temperature, humidity and evaporation data are lacking entirely, as was also challenging in other studies [27]. This uncertainty affects the calibration process of comparing the known record of an environmental variable to the tree-ring chronology for the purpose of determining tree growth response [109].

A low common signal has been observed for some other tropical trees. Ref. [124] found ring width to be highly variable between different radii of the stem, with low correlations among individual trees for six tropical Bolivian species. *J. australis* has a similar mean within-tree correlation of radii (0.68–0.70) to those found in our study (0.760), but *J. australis* shows a higher among-tree correlation (0.449–0.503) than *J. neotropica* [29].

Wood formation in a given year is dependent partly on growing conditions in the prior year [153,154]. Growth of *J. neotropica* is influenced by precipitation during previous and current growing season [27], similar to studies of *J. australis* [103]. Worbes’ [105] study of 37 tropical tree species in a semi-deciduous forest in Venezuela found that evergreen species tended to have a short growth interruption (during the latter part of the dry season), whereas deciduous species stopped growth completely at the end of the rainy season. Our findings of annual bands in *J. neotropica* suggest a complete cessation of growth during the dry season in this environment. Consistent with our conclusions, ref. [27] found that tree-ring chronologies were most highly correlated with the precipitation period from November (year *t*) to October (year *t* + 1).

### 4.2. Are Ring-Based Age Estimates Consistent with Radiocarbon and Stable Isotopic Estimates?

The radiocarbon result and the visual dating of JN37-1W yielded different age estimates of up to ±five years (±12%) (Table 2). This difference in age interpretation is within the uncertainty of the respective age models but may be partly due to undetected dating errors in tree sample JN37-1W, due to surface irregularities that remained because we wished to avoid sample cross-dust contamination in isotope and radiocarbon processing [126]. As emphasized earlier (e.g., Figure 3D), annual rings can be difficult to detect visually if not sanded. Oxygen isotopic results at intraseasonal resolution suggest a quasi-cyclical climatic signal (Figure 4, middle), which, if interpreted as variation over the course of the growing season, produces a chronology consistent (within uncertainty) with independently derived radiocarbon and ring width age models.

All three of the dating methods used in this study are imperfect and involve uncertainties. The cross dating of tree rings produced chronologies with a low signal-to-noise-ratio and may also have been influenced by the relative youth of the sampled trees. Although the derived signal appears to reflect quasiperiodic annual cycles, oxygen isotopic analyses are expensive and time consuming to replicate. Radiocarbon dating uncertainties arise from dendrochronological uncertainty, and the bimodal distribution of the peak atmospheric radiocarbon concentrations with latitude, and measurement uncertainties [155,156,157,158]. Despite these uncertainties, results from all three chronological methods are convergent, and provide a more coherent picture of the extent to which precipitation variations may be reflected in proxy observations than might be derived from a single approach [25].

This study revealed that the *Juglans* collection from northern Piura is probably not more than 45 years old. This conclusion is consistent across all proxy measurements employed (tree-ring chronology, isotope age interpretation and radiocarbon results). Considering the challenges in working with tropical tree species, our results are encouraging for the potential of tropical dendroclimatology. Our investigation suggests useful implications for climate reconstruction, sustainable forestry and carbon cycle dynamics in the dry forests of tropical South America. Human use of *J. neotropica* is intensive in the study area, which emphasizes the importance of long-term planning and protection to maintain sustainable populations of the species.

### 4.3. Are Variations in Ring Widths, Growth Rates and Oxygen Isotopic Composition of J. neotropica at Piura Related to Regional Climate and ENSO, and If So, How?

Growth modeling results are consistent with our expectation that tree growth in *J. neotropica* is conditioned primarily on growing season precipitation, not temperature (Supplementary Information, Figure S3). A growth lag response of one year of *J. neotropica* with respect to local and regional precipitation was evident in some periods in our standard growth curve, such as 1973/74, 1987/88, 1989/90 and 1995/96 (Figure 4, top). Ref. [159] proposed the ‘pulse-reserve hypothesis’ to explain how arid and semi-arid plants respond to trigger events (e.g., precipitation events) versus time. He argued that the response to a sequence of rain events depends in part on the time interval between events relative to the ‘relaxation time’ of the system (dry periods) in response to individual events. If rain events are clustered or the plant has a slow response, the effects of the input pulses (rain) will accumulate to produce one single response pulse (growth). This response will often be delayed in time, which can be manifested as a lagged response in the *J. neotropica* growth curve, such as 1973/74, 1987/88, 1989/90 and 1995/96 (Figure 4, top). Correlation analysis for *J. neotropica* during the period 1973/74–2005/06 indicates a significant positive relationship between ring width lagged one year behind Santo Domingo precipitation (*r* = 0.47, *p* = 0.004, edf = 16). We also found a positive (but not significant) relationship between ring width lagged with respect to Frias precipitation by one year (*r* = 0.32, *p* = 0.10, edf = 8.5). These relationships could be explored more fully with larger sample sizes and longer time series; differences in correlation may result from real spatial variations in precipitation anomalies, chronological uncertainty, and meteorological data uncertainty. A growth lag response of one year of *J. neotropica* with respect to gridded precipitation in Peru was also found by [27].

Because of the significant positive correlation between Santo Domingo precipitation and ring width growth, and the marginally significant correlation with Frias precipitation, we tentatively reject the null hypothesis that wood growth of *J. neotropica* is independent of precipitation. Investigations of *Prosopis* spp. in northern Peru report a significant relationship to precipitation events from October the previous year until June of the current year [44]. Our interpretation is consistent with [103], who found that growth in several tropical tree species (including *J. australis*) was associated positively with precipitation, although there were differences in the timing of the response between species: some species showed a strong positive relationship to precipitation during the previous growing season, while other species showed a positive relationship only to current growing season precipitation, and others responded to both current and previous growing season precipitation. Understanding the mechanisms of a lagged growth response to environmental variability would be an important direction for further research with these species.

Increased ring width growth is consistent with anomalously high precipitation resulting from the strong El Niño events of 1982/83, 1991/92, 1997/98 and 2003-05. Refs. [105,160] found similar correlations between El Niño events and wood growth in tropical tree species. The El Niño events of 1982/83 and 1997/98 produced five and seven times the mean of annual precipitation in Frias and Santo Domingo, respectively; tree-ring growth for these years increases to local maxima during those years compared to the previous and following years, but 1972/73 and 1982/83 seems to precede the appearance of new cohort of trees in the sample (Figure 4, top and Figure 5a,c). The moderate growth increase associated with the El Niño events of 1982/83 and 1997/98 may be due to water saturation (i.e., too much water for plant utilization in a short time period). The strong El Niño event of 1973/74 seems to show a lag effect of one year (Figure 4, top) and appears to be explained by clustered rain events in previous years and/or a slow response of the species. This is consistent with the results of [103], showing that growth variations of *Juglans australis* were controlled largely by precipitation during previous and current growing seasons. Our results are also consistent with *Bursera graveolens* [42] and *Prosopis pallida* [44,45] in northern Peru, which show increased tree ring growth in response to El Niño events.

Although we found that *J. neotropica* responds to the previous season’s precipitation, this effect seemed to be moderated during especially high precipitation periods, such as strong El Niño events. The threshold–delay (T–D) model [161] in arid and semi-arid environments proposes that antecedent water availability affects a plant’s ability to use precipitation [162,163]; this effect can be incorporated into the lag or threshold response, such as the precipitation thresholds, for which plant response is reduced. This model might account for the immediate growth response in the tree-ring chronology related to some large precipitation events, whereas a lag occurs in years of less precipitation (Figure 5).

The significant negative correlation between gridded precipitation and isotopic composition supports the hypothesis that isotopic composition in *J. neotropica* is controlled by the regional precipitation amount, itself influenced by ENSO variation [72]. Isotopic age interpretations of JN37-1W and JN41-2N showed a response to precipitation; the two El Niño events of 1982/83 and 1997/98 are manifested as a ~−3‰ magnitude variation in δ^18^O_cellulose_ in both age interpretations, most clearly in JN41-2N (Figure 4, middle). Ref. [15] found that *Prosopis pallida* in Piura can respond to El Niño events with a similarly large magnitude variation in δ^18^O_cellulose_ [65] found that the variation in cellulose δ^18^O of cloud forest trees was consistent with both simulated δ^18^O and anomalous winds and sea surface temperatures associated with the footprint of ENSO in the tropical Atlantic and Pacific. Our findings support [81], who suggested that large-scale atmospheric circulation features should be considered when interpreting tree ring oxygen isotope signals, because circulation represents not only large-scale processes fundamental to source water δ^18^O, but also the extent of enrichment due to evapotranspiration in the leaf.

We found a significant relationship between tree-ring growth lagged by one year relative to local precipitation, whereas the isotope interpretation showed a significant relationship to gridded precipitation and simulated isotopic composition in the same year. Considering the biological facts of cell processing in a tree, compared with isotopic theory in dendrochronology, we expected a similar response between the two proxies, because the isotopic composition of water is primarily recorded in new cells, as part of the growth of the tree (but see [164], who proposed and modeled a carry-over mechanism from the prior year’s stored photosynthate). It is well established that antecedent rainfall affects tree growth in arid and semiarid environments [159,161,165], and that mixing processes and lag-times in the soil may vary from year to year and thus influence the tree-ring signal [81]. Many questions remain unanswered and need further investigation in this ecosystem, but our study suggests the merits of using ring width and isotopic data in combination to further understand the environmental information encoded in trees, compared to either measurement in isolation. Unlike other studies (e.g., [25], review by [72]), the oxygen isotopic data in this study were age modeled independently of cross-dating, using their interannual cyclicity and also radiocarbon data, with subsequent environmental interpretation of their interannual variations. Such a strategy might be useful in future studies of *J. neotropica* and other species from our study region, where ring-width cross-dating is uncertain.

Low replication of the observations presented here is a valid limitation on the strength and generality of the interpretations we develop. Although we found a significant correlation between seasonal δ^18^O and gridded precipitation for 1973/4–2001/2002, gridded precipitation data were not significantly correlated with isotope interpretations of current or previous seasons for 1959/60–2005/2006. This is likely to reflect the lack of a two-sample composite prior to 1978/9, and that oxygen isotope age model error is cumulative and increases with time since present [15,85,130]. If these preliminary correlations are valid, then improved age model uncertainty with additional replicate analyses should improve ring width chronology statistics (Table 1), and the significance of correlation of isotopic data with gridded regional precipitation, modeled cellulose δ^18^O and large-scale climate variations (Table 4).

Lower replication records, such as those presented here, have been shown repeatedly to be of value, at least as preliminary indicators. With respect to cellulose δ^18^O records, [69] published interpretable and statistically significant correlations simulated with four cores from three trees. [65] used two radiocarbon-dated increment cores to form an age model with *n* = 2 replication for 1900–1969, with significant r across the two records, and significant correlation with environmental variation associated with ENSO activity. [25] averaged δ^18^O data from three cores from three trees to show significant precipitation-related correlation with ENSO activity at a site in Queensland, Australia. [131] averaged data from three cores from three trees for the period 1971–2005, and showed significant correlation with both local precipitation amount, itself a local response associated with remote ENSO activity. That interpretation has recently been replicated independently by correlation of an expanded composite δ^18^O record with NINO34 SST anomaly, 1856–2005 [166].

## 5. Conclusions

*Juglans neotropica* has annual rings and a dormant period during the dry season of the annual year cycle; we developed a ring-width chronology for this species in a semi-arid, mid-elevation environment at ≤5 °S. This study also demonstrates that variation in δ^18^O_cellulose_ and radiocarbon analyses provide complementary and independent chronological control. Both ring width and oxygen isotopic composition provide consistent and plausible indicators of the precipitation variation most clearly associated with interannual ENSO warm phase events.

The age of the *J. neotropica* sample in this study is young, probably as a consequence of human exploitation of the species, but the techniques applied here may be applied to other species, including some with greater longevity. Such studies might be used to test the hypothesis that El Niño events can initiate changes in stand demography. The combination of wood anatomy, tree-ring chronologies, radiocarbon analyses and stable isotopic analyses provide a greater degree of confidence in our chronological results that would have been possible using only a single proxy. Increased sample replication, geographic range, and more controlled study of the environmental controls on *J. neotropica* growth and wood isotopic composition would be beneficial to fully exploit the dendrochronological potential of this species to record aspects of climate and carbon cycle dynamics in the dry forests of tropical South America [167].

This study contributes to the increasing inventory of tree-ring chronologies near the equator and in montane areas of South America. The expanded study of tropical forests has the potential to illuminate how these ecosystems influence the dynamics of the global carbon cycle, as well as deeper insights into how climate variability modulates tree growth, survival [168,169], and reproduction in tropical forest ecosystems. Tropical dendrochronology has much to contribute to insights into climate and forest dynamics, both regionally and globally.

## Figures and Tables

**Table 1:** Chronology statistics for *J. neotropica*.

**a. Chronology Time Span: 1963 to 2006 (44 Years), 40 Trees, 112 Radii**	
**Chronology Type**	**Standard:**
Mean	0.9590
Median	0.9670
Mean sensitivity	0.1539
Standard deviation	0.1705
Skewness	0.7399
Kurtosis	2.1267
Autocorrelation order 1	0.3703
Partial autocorr order 2	0.1011
Partial autocorr order 3	−0.4864
**b. Common Interval Time Span: 1977 to 2006 (30 Years, 17 Trees, 28 Radii)**	
**Mean Correlations:**	**Detrended Series:**
Among all radii	0.023
Between trees (Y variance)	−0.007
Within trees	0.760
Radii vs. mean	0.176
Signal-to-noise ratio	−0.124
Agreement with population chron	−0.142
Variance in first eigenvector	23.83%
Chron common interval mean	0.954
Chron common interval std dev	0.121
*r*-bar	0.176

**Table 2:** Radiocarbon results (^14^C) of five samples from *J. neotropica,* radius JN37-1W.

Sample # (Distance Along Core from Bark in cm)	F ^14^C (from AMS Lab)	Uncertainty in F^14^C	d ^13^C (from AMS Lab)	Calibrated Recruitment Year Range at 95.4% Certainty	Expected Recruitment Years	Range of Years in Difference Between Expected and Calibrated Recruitment Year
1 (19.1)	1.1238	0.0064	−27.6	1956–1961	1966–1967	5–11
2 (16.9)	1.2426	0.0095	−26.1	1961–1966	1969–1970	3–9
3 (15.3)	1.5940	0.0100	−25.9	1963–1969	1972–1973	3–10
4 (9.4)	1.4080	0.0080	−27.3	1980–1986	1981–1982	1–5
5 (6.5)	1.0943	0.0063	−26.0	1991–1998	1990–1991	0–8

## Data Availability

The original contributions presented in the study are included in the article and Supplementary Material. Tree-ring width and isotopic data collected for this study will be available through the NOAA/NCEI World Data Service (https://www.ncei.noaa.gov/products/paleoclimatology). Further inquiries can be directed to the authors.

## Supplementary Materials

The following supporting information can be downloaded at: https://www.mdpi.com/article/10.3390/plants14111704/s1. Figure S1: Banded age model (BAM) simulation of age uncertainty of tree-ring width (upper panel; 5% error rate) and δ18O (lower panel; 5% error rate.; Figure S2: Correlation of plausible age simulated TRW (left) and δ18O (right) series with the NINO12 (upper) and NINO34 SST (lower) anomaly indices.; Figure S3: VS-Lite modeling of temperature and moisture controls on growth. Relative effects of monthly temperature (GT, upper left) and moisture (GM, upper right) on TRW. Resulting simulated TRW time series with propagated age model errors (lower left), and comparison of simulated and observed TRW (lower right).
