# Supplementary material for: Dendrochronology and Isotope Chronology of Juglans neotropica and Its Response to El Niño-Related Rainfall Events in Tropical Highlands of Piura, Northern Peru
Source: Plants (Basel). 2025 Jun 3;14(11):1704. doi: 10.3390/plants14111704 (PMC12158084; doi:10.3390/plants14111704)

Supplemental Information

Both ring width and oxygen isotopic data reported in this study have considerable chronological uncertainty. We used the banded age model (BAM) of Comboul et al (2014) to realistically simulate the effect of age uncertainty on correlation analyses reported in this work. Using published code (https://github.com/CommonClimate/common-climate), we specify a Poisson distribution for dating errors that occur randomly as missing or additional annual increments and accumulate back in time. We specify an error rate of 5% for ring widths and 10% for the $\delta^{18}O$ data. The results are shown in Figure S1 and visualize 1000 realizations of the resulting age uncertainty.

Figure S2 displays the correlation of these sets of plausible age simulated TRW and $\delta^{18}O$ series with the NINO12 and NINO34 SST anomaly indices. The results are 95th percentile confidence intervals of [0.04, 0.37] and [-0.39, 0.44] for correlation between NINO12 and TRW and $\delta^{18}O$, respectively, and CI of [-0.07, 0.28] and [-0.18, 0.44] for correlation of NINO34 with TRW and $\delta^{18}O$, respectively. Modes of those correlations are 0.34 and -0.35 for correlation between NINO12 and TRW and $\delta^{18}O$, respectively, and 0.09 and -0.10 for correlation between NINO34 and TRW and $\delta^{18}O$, respectively.

Tree-ring growth modeling

VS-Lite (Tolwinski-Ward et al 2011) is a plausibly realistic multivariate and nonlinear model simulating tree ring width, based on the principle of limiting factors, and requiring a relatively small set of inputs and parameters (Tolwinski-Ward et al 2011; Evans et al. 2014). In this study, we used VS-Lite to identify plausible model parameters that would simulate TRW consistent with the ensemble of age modeled series shown in Figures S1 and S2.

We conditioned threshold temperature (T_1, T_2) and soil moisture-related (M_1, M_2) growth parameters on the fit of TRW simulations to observations over the period 1973-2001 (r = 0.31, edf = 8, p = 0.07). Rooting depth was set to 3.5 m, consistent with observations of this species from Toro and Roldán (2018). We specified all other modeling parameters as default values (Tolwinski-Ward et al. 2011). Forcing was provided by CRU TS4.08 nearest grid point air temperature and precipitation (Harris et al. 2020). Analysis of intra-annual temperature (G_T) and moisture (G_M) growth functions (Figure S3, upper left and right panels, respectively) show that the results are consistent with moisture (thick black line), rather than temperature,

being the limiting factor determining observed TRW, and environmental limitations to overall growth $G = G_S*min(G_T,G_M)$ (thick blue line) during December-January.

Figure S1. Banded age model (BAM) simulation of age uncertainty of tree-ring width (upper panel; 5% error rate) and $\delta^{18}O$ (lower panel; 5% error rate.

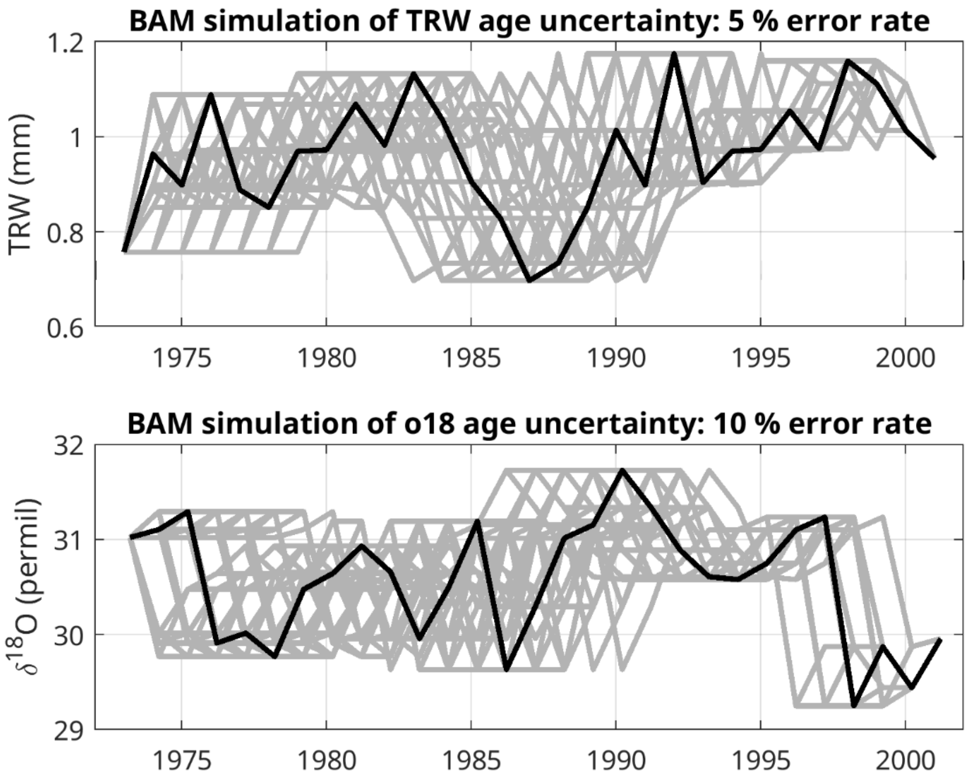

Figure S2. Correlation of plausible age simulated TRW (left) and $\delta^{18}O$ (right) series with the NINO12 (upper) and NINO34 SST (lower) anomaly indices.

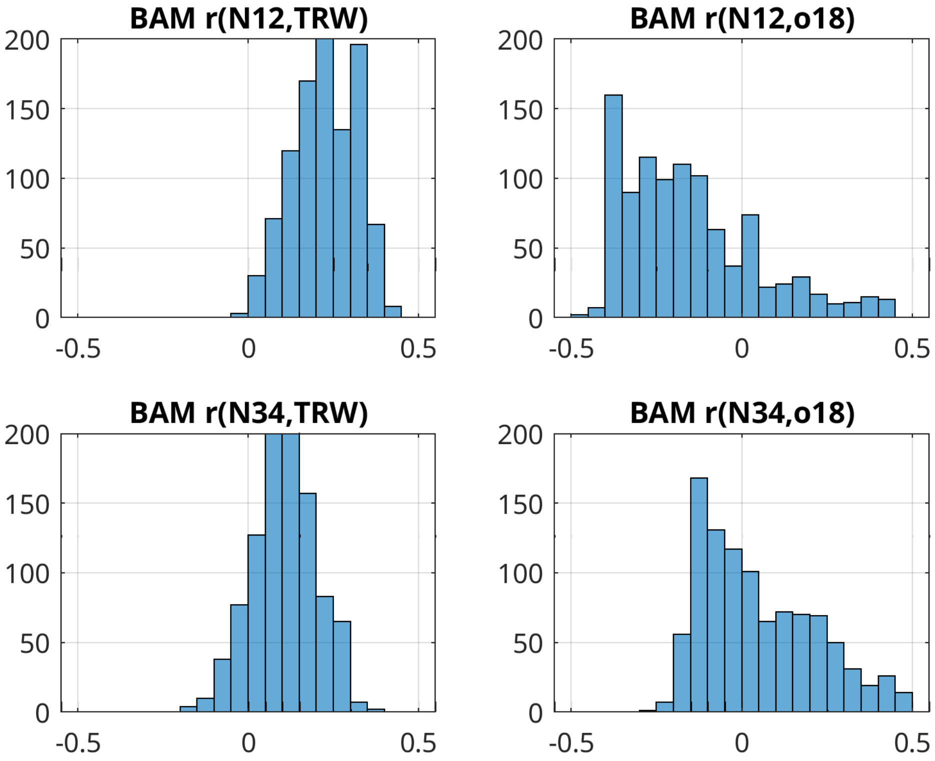

Figure S3. VS-Lite modeling of temperature and moisture controls on growth. Relative effects of monthly temperature (G_T, upper left) and moisture (G_M, upper right) on TRW. Resulting simulated TRW time series with propagated age model errors (lower left), and comparison of simulated and observed TRW (lower right).

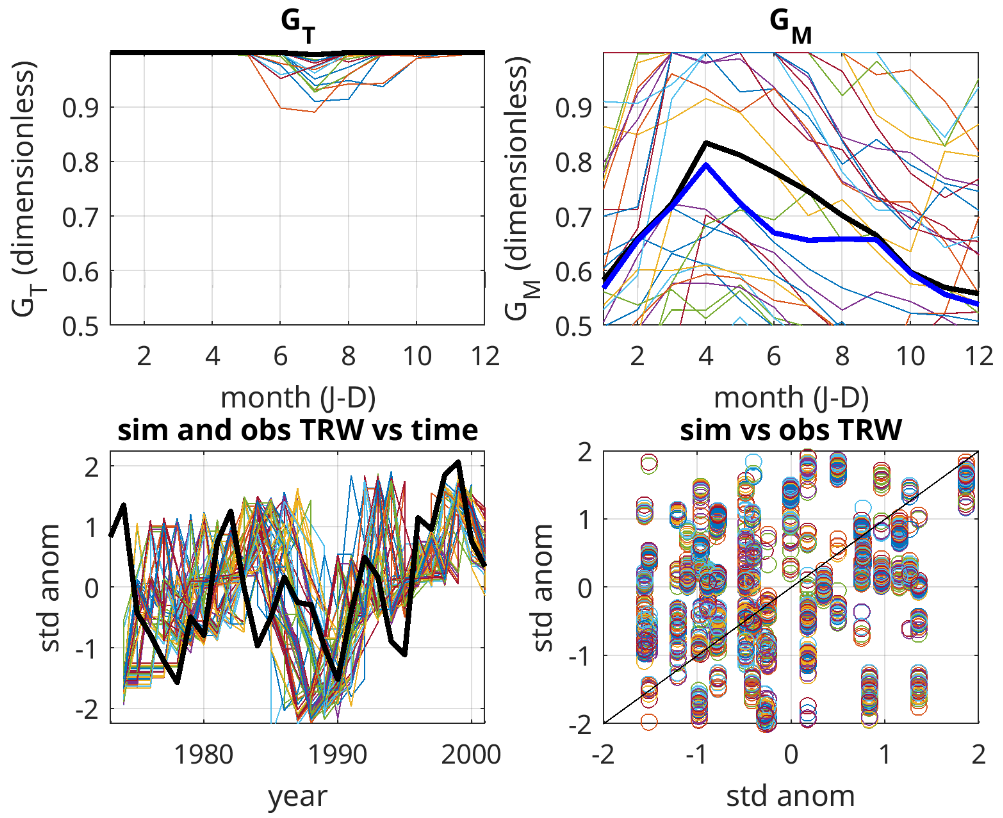

Supplement: Supplementary file 1 [file plants-14-01704-s001.zip › plants-3590539-supplementary.pdf]
